# Intratumoral heterogeneity of endogenous tumor cell invasive behavior in human glioblastoma

**DOI:** 10.1038/s41598-018-36280-9

**Published:** 2018-12-20

**Authors:** Jonathon J. Parker, Peter Canoll, Lee Niswander, B. K. Kleinschmidt-DeMasters, Kara Foshay, Allen Waziri

**Affiliations:** 1 Stanford University School of Medicine, Department of Neurosurgery, Stanford, CA USA; 2 Columbia University College of Physicians and Surgeons, Department of Pathology and Cell Biology, New York, NY USA; 3 University of Colorado, Department of Molecular, Cellular & Developmental Biology, Boulder, CO USA; 4 University of Colorado School of Medicine, Department of Pathology, Anschutz Medical Campus, Aurora, CO USA; 5 University of Colorado School of Medicine, Department of Neurosurgery, Anschutz Medical Campus, Aurora, CO USA; 6 Inova Neuroscience and Spine Institute, Inova Health Systems, Falls Church, VA USA

## Abstract

Intratumoral genetic heterogeneity is a widely accepted characteristic of human cancer, including the most common primary malignant brain tumor, glioblastoma. However, the variability in biological behaviors amongst cells within individual tumors is not well described. Invasion into unaffected brain parenchyma is one such behavior, and a leading mechanism of tumor recurrence unaddressed by the current therapeutic armamentarium. Further, providing insight into variability of tumor cell migration within individual tumors may inform discovery of novel anti-invasive therapeutics. In this study, *ex vivo* organotypic slice cultures from EGFR-wild type and EGFR-amplified patient tumors were treated with the EGFR inhibitor gefitinib to evaluate potential sub-population restricted intratumoral drug-specific responses. High-resolution time-lapse microscopy and quantitative path tracking demonstrated migration of individual cells are punctuated by intermittent bursts of movement. Elevation of population aggregate mean speeds were driven by subpopulations of cells exhibiting frequent high-amplitude bursts, enriched within EGFR-amplified tumors. Treatment with gefitinib specifically targeted high-burst cell subpopulations only in EGFR-amplified tumors, decreasing bursting frequency and amplitude. We provide evidence of intratumoral subpopulations of cells with enhanced migratory behavior in human glioblastoma, selectively targeted via EGFR inhibition. These data justify use of direct human tumor slice cultures to investigate patient-specific therapies designed to limit tumor invasion.

## Introduction

The innate ability of glioblastoma to infiltrate normal brain is a clinical challenge, which limits efficacy of surgical resection, radiotherapy, and chemobiotherapies. Consortium based efforts utilizing large-scale data analyses reveal extensive GBM heterogeneity at the inter-tumoral level, and several molecular subtypes have been defined based on commonly observed genetic and epigenetic changes^1,2^. While detection of *IDH1* mutation and/or methylation of the *MGMT* promoter are now correlated to increased overall survival^3–6^, the prognostic value of other common genetic mutations, including amplification of the *EGFR* locus, remains unclear^7^. The ability to understand the network of connections between genetic heterogeneity, tumor cell phenotype, and disease progression, has potential to improve therapeutic targeting via increasing accuracy of predictions of drug response.

Our lab recently demonstrated phenotypic heterogeneity in GBM migratory potential, which correlates to patient-specific *EGFR* amplification status. Amplification at this locus, which is detected in 40–50% of GBM tissues^8^, is typically mosaic and believed to enhance pro-invasive signaling through EGFR. Interestingly, clinical imaging suggests this subset of receptor-amplified cells is enriched at the infiltrative tumor edge^9,10^. Supporting these data, our *ex vivo* slice cultures demonstrated increased tumor cell migration in *EGFR*-amplified tumors, and blockade of EGFR signaling with the small molecule inhibitor, gefitinib, induced a statistically significant reduction in migratory behavior within the same sample set^11^. Identification of this link between genetic heterogeneity and tumor cell behavior provides a paradigm for the mechanistic link between molecular variation and cellular behavioral changes which dictate therapeutic response.

Despite evidence supporting the pro-migratory role of EGFR in GBM progression, trials of gefitinib and other targeted receptor tyrosine kinase (RTK) therapies, alone or in combination, failed to extend patient survival^12^. This disconnect between *in vitro* drug studies and *in vivo* efficacy led the field to consider the prevalence of molecular heterogeneity within individual tumors as a mechanism of treatment resistance. Integrated analysis of primary GBM revealed significant gene expression changes within samples isolated from different regions of the same tumor^13^. These findings were confirmed at the cellular level through single-cell RNA-seq, which identified cell-to-cell variation in regulation of growth, metabolism, and immune response trasncripts^14^. More recently, single-cell sequencing highlighted differential expression in cells of the tumor core as compared to those of the infiltrated penumbra^15^.

To date, the extent to which genetically or epigenetically distinct subsets of cells, present within individual human GBM tumors, contribute to overall variation in cell behavior and drug response *in vivo* remains unclear. However, in the PDGF-driven rat glioma model, two distinct tumor-associated cell populations exhibit disparate migratory potentials in response to PDGF secretion, suggesting that a particular sub-population can dominantly contribute to the invasiveness of the tumor, as a whole^16^. Indeed, differential amplification of RTKs, including EGFR, PDGFR, and MET was observed within tumor cells isolated from distinct regions of multifocal GBM in individual patients^9,17,18^. Our previous studies utilized low-resolution path-tracking that was sufficient to detect inter-patient but not intratumoral migrational heterogeneity. We hypothesize that intratumoral molecular heterogeneity may manifest as measurable differences in migratory potential within human GBM cell subpopulations.

In the current study, we perform high temporal resolution path-tracking analysis to gain insight into the divergence of migratory behavior within individual tumors. We demonstrate the presence of small, fast moving subpopulations of cells that dictate overall tumor invasiveness. Interestingly these fast cells are more prevalent within *EGFR*-amplified tissues and, preferentially respond to gefitinib, emphasizing the clinical implications of intratumoral heterogeneity. Whether molecularly distinct subpopulations arise early in disease progression, or from treatment-resistant cells, their presence necessitates consideration in predicting response to, and failure of, targeted therapeutics.

## Results

### Human glioblastoma slice cultures reveal intratumoral variation in cell migration

**Figure 1:**
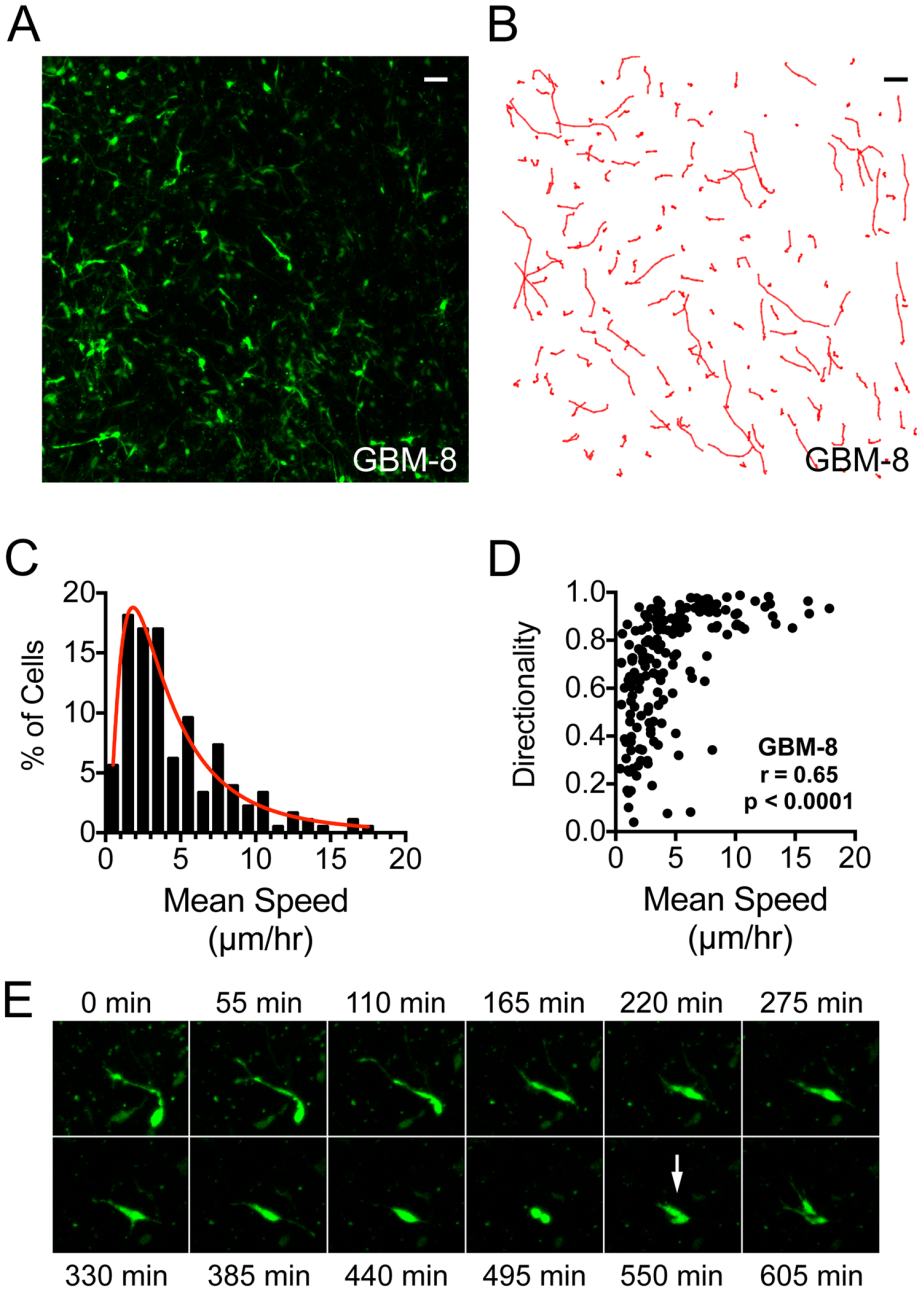
High temporal resolution path tracking highlights intratumoral heterogeneity of speed and directionality of tumor cell migration in focal micro-regions. (A) 10x field view of tumor cells in GBM-8 labeled by transduction of an MMLV-retroviral vector constitutively expressing ZsGreen, and (**B**) accompanying migration paths after 11 hours of imaging. Scale bars represent 50 μm. Tumor cell location was tracked every 11 minutes. Cells with long, direct paths are observed migrating past stationary cells. (**C**) Cell speeds are distributed log-normally (R^2^ = 0.95) in this representative population (GBM-8). The red line represents the best fit regression to a log-normal base model. (**D**) Correlation between directionality and speed in a representative tumor (GBM-8, r = 0.65, p < 0.0001). Similar results were obtained in 5 out of 7 tumors analyzed (Spearman r correlation coefficient ranged from 0.45 to 0.65). (**E**) A representative tumor cell tracked over 10 hours, in 55-minute intervals, demonstrating rapid cell division during migration (GBM-13), coupled with the instantaneous speed over migration time for this cell (right).

While cell-to-cell variation in gene expression and receptor tyrosine kinase amplification within GBM are well recognized, it remains unclear how genetically and epigenetically distinct subpopulations contribute to variation in cell behavior. We hypothesize that intratumoral molecular heterogeneity manifests as quantifiable variation in migratory behavior within the tumor microenvironment. Indeed, our previous analysis of 20 human GBM tumors revealed inter-patient differences in cell migration and highly variable migration patterns within individual tumors, suggesting intrinsic heterogeneity in migratory potential from cell to cell (Fig. 1A,B)^11^.

To assess patterns of migration in individual cells, we tracked cell movement over 11 hours using time-lapse confocal microscopy of human GBM slice cultures. Proliferative cells within the tumor were preferentially labeled by transduction with an MMLV-retroviral vector constitutively expressing ZsGreen. Migrating tumor cells moved intermittently, pausing to dynamically probe the environment with filopodia, followed by bursts of rapid movement mediated by filopodia retraction and nuclear deformation. This saltatory pattern of movement was previously described in neural progenitors cells, GBM xenografts, and PDGF-driven GBM mouse models, suggesting it may be intrinsic to GBM invasion^19–23^. Notably, migratory cells moved at high speeds passing stationary cells, which did not move over the imaging time frame, resulting in single-cell migration speeds that varied widely. When represented graphically, mean speeds approximate a log normal distribution with a long tail suggestive of a small subpopulation of fast migrating cells (Fig. 1C).

In addition to speed, a cell’s directionality, described by the ratio of cell displacement over total distance traveled, contributes to migration efficiency. Time-lapse microscopy revealed cell-to-cell variability in directionality, and in 5 out of 7 tumors analyzed we found a significant positive correlation with mean migration speed (Spearman r ranged 0.45 to 0.65, mean 0.56) (Fig. 1D, GBM-8 shown). Thus, the fastest migrating tumor cells are also the most efficient in their tissue-traversing path. These data confirm the existence of cell-to-cell heterogeneity in patterns of migration with dynamic evidence shown for the first time within patient-derived living GBM tissue.

While the observation of intratumoral migratory heterogeneity in human GBM is novel, tissue level spatial variation in proliferative behavior has been reported around histological structures^24^. While PDGF-driven mouse models suggest tumor cells simultaneously maintain high rates of proliferation and migration^23^, the “go or grow” hypothesis suggests mitotically active tumor cells are less migratory^25^. Thus, we used time-lapse images of active cell division to evaluate mitotic variation as one plausible explanation for observed migrational heterogeneity in our slice model (Fig. 1E). Dividing tumor cells paused only transiently from their migratory program, retracted filopodia, completed mitosis, and re-initiated migration in less than 3 hours (Fig. 1E). Cell division is less prevalent in human tumor slice cultures as compared to mouse models, but observed mitotic events were not restricted spatially, and recurrent divisions in individual cells were not observed during the imaging period. These data suggest cell division does not explain the “pause and burst” pattern of movement, or the widespread intratumoral heterogeneity observed with respect to migration speed.

### Bursts of tumor cell movement contribute to increased overall migration speed

Our studies confirmed significant heterogeneity in the mean speed and directionality of cells migrating within GBM tissue. Given the “pause and burst” nature of tumor cell migration, we postulated that the frequency and amplitude of bursts, may contribute significantly to overall cell speed. Indeed, others have suggested that high speed movements are predictive of migratory behavior over long cell tracks^26^ and of overall tumor invasiveness^22^.

**Figure 2:**
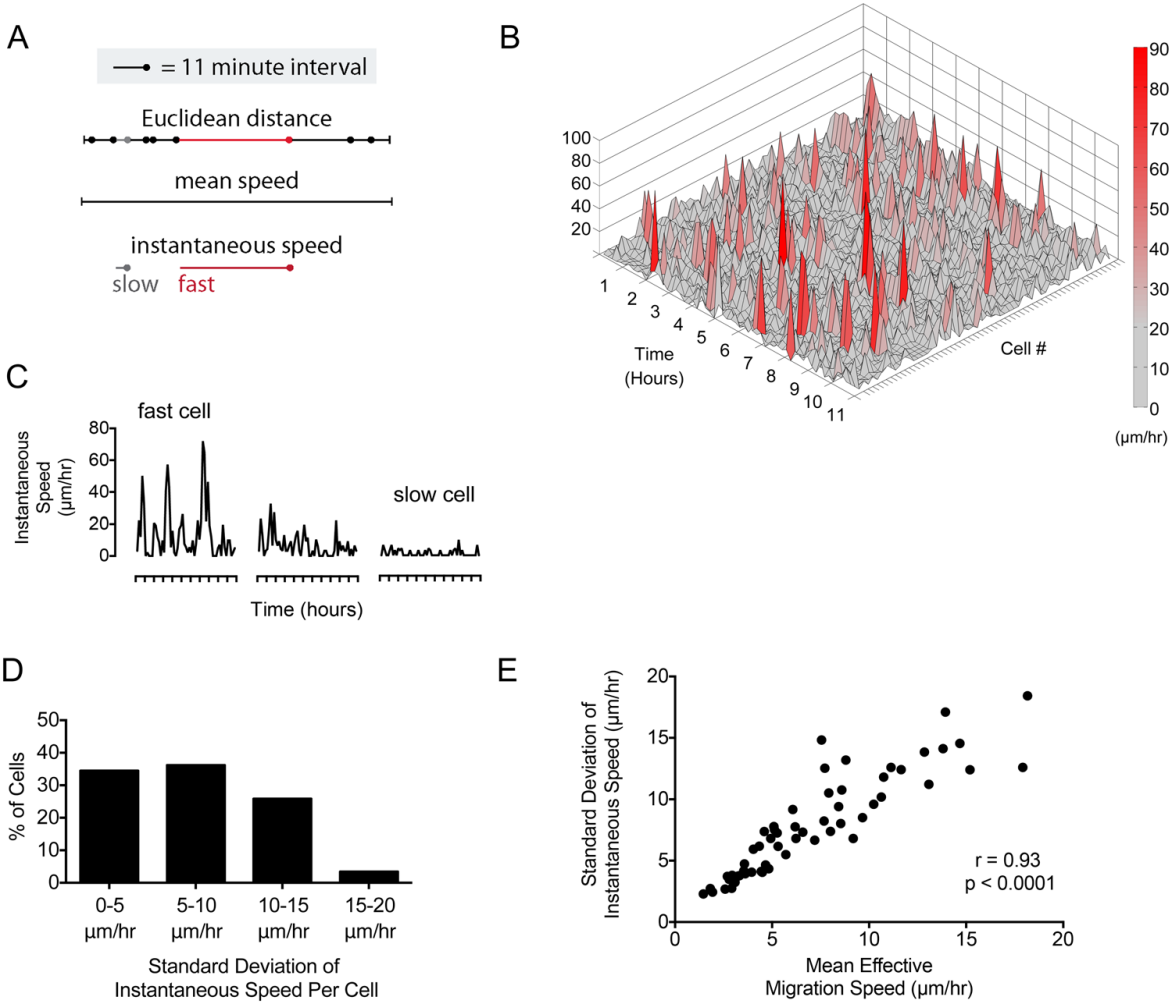
Surface topography analysis highlights a subpopulation of glioblastoma cells with increased bursting activity. (**A**,**C**). Line based schematics of differences in Euclidean distance traversed per unit time during a burst of cell migration (red line). (**B**) Surface topography map demonstrates bursting behavior of cells from a representative tumor cell population (GBM-8 shown, analysis performed in all tumors n = 7). Peak height represents speed (μm/hr) with a color-coded heat map (right). (**D**). Cells with frequent bursting behavior (increased standard deviation of instantaneous speed) represent a small subpopulation within the tumor. (**E**) There is a positive correlation between standard deviation of instantaneous speed and mean effective speed for individual cells (Spearman correlation coefficient, r = 0.93, p < 0.0001).

To explore variation in bursting behavior we performed high temporal resolution tracking of tumor cells in our cohort of GBM slice cultures (n = 7, Supplemental Fig. 1C). The instantaneous speed of each cell was calculated utilizing high granularity tracking, every 11 minutes, and plotted in three dimensions to render a surface topography map (Fig. 2A,B). Within each tumor analyzed, we noted high-amplitude peaks that represent bursts of migration much faster than “baseline” migration speed. Some cells had numerous high amplitude peaks, while other cells had few or none (Fig. 2C and Supplemental Fig. 1A,B). This variability, or oscillation from “baseline”, can be represented by the standard deviation of the instantaneous speed. Cells with the greatest standard deviation in instantaneous speed, or the largest bursts of movement, represented less than 5 percent of the total population (Fig. 2D). Among these cells, there was a positive correlation between standard deviation of instantaneous speed and effective migration speed of the cell (r = 0.93, p < 0.0001) (Fig. 2E). These data confirm that the bursting behavior of a small population of fast cells within the tumor contributes to effective migration and overall tumor speed.

### *EGFR* amplification contributes to increased intratumoral heterogeneity with respect to cell migration

**Figure 3:**
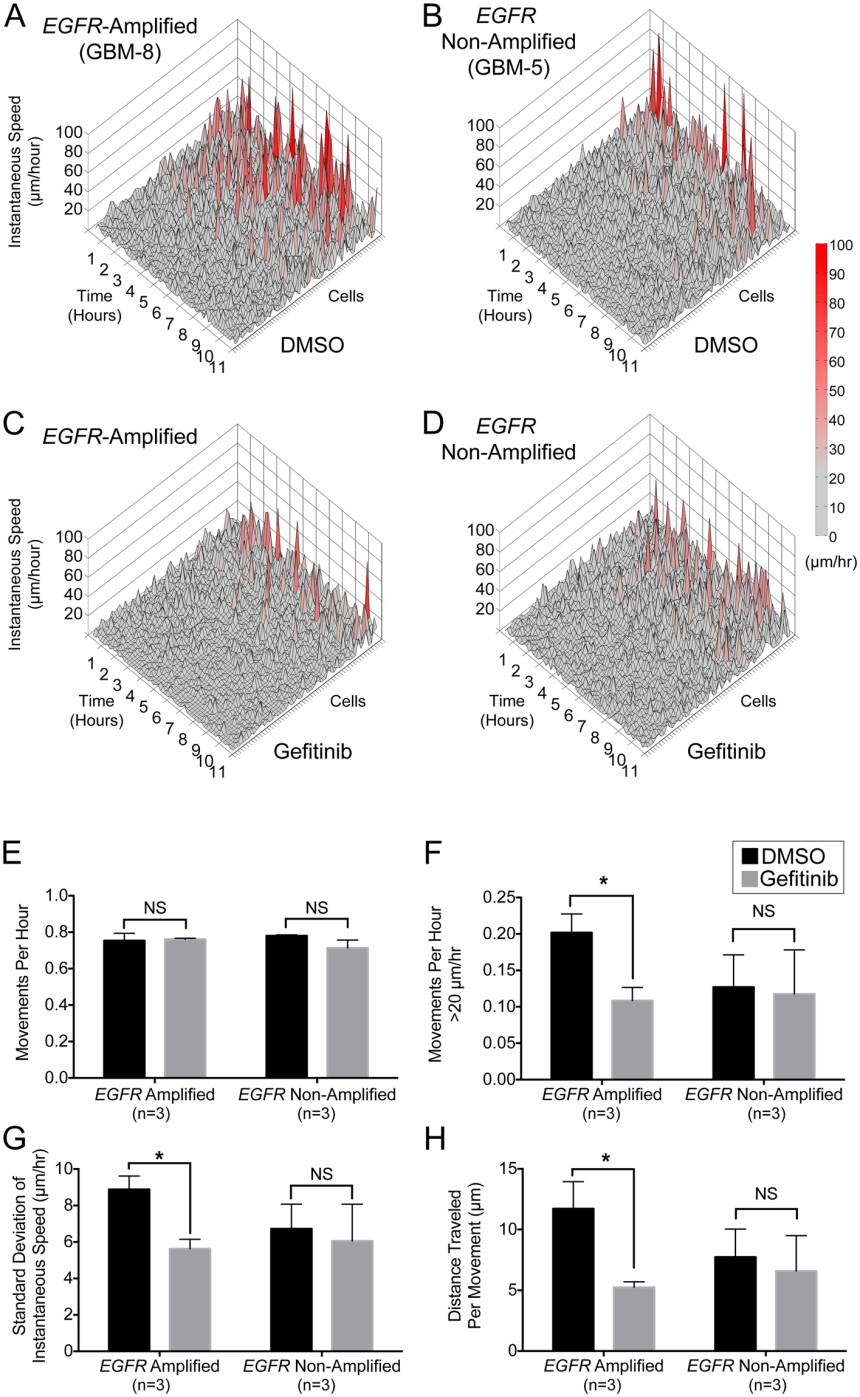
Gefitinib treatment disrupts high-speed burst behavior selectively among tumor cell populations within *EGFR*-amplified glioblastoma. (**A–D**). Topography graphs of instantaneous tumor cell speed over time, where rows represent individual cells. For all treated samples (n = 6) cells were tracked every 11 minutes and ordered by increasing standard deviation of instantaneous speed. Peak height and color-coded heat-map shading both represent cell speed, representative data from GBM-5 and GBM-8 are shown. Gefitinib treatment (10 μM) for 11 hours, resulted in a qualitative decrease in high-amplitude peaks in an *EGFR*-amplified slice (GBM-8, A,C). Non-significant changes were observed after treatment of a non-amplified tumor slice (GBM-5, B,D). (**E**) Movements per hour (i.e. peaks/hr) did not change between control (DMSO, black bars) and gefitinib (grey bars) treatment in pooled cell populations from all *EGFR*-amplified or non-amplified tumors (p > 0.05, n = 3 amplified tumors, n = 3 non-amplified tumors). (**F–H**) High-speed movements, standard deviation of instantaneous speed, and distance traveled by the cell per movement were significantly decreased in *EGFR*-amplified tumors (n = 3, p < 0.05), with no effect in non-amplified tumors (n = 3, p > 0.05). Error bars represent standard error of the mean (SEM).

Genomic amplification of the WT EGFR receptor is common in GBM and displays intratumoral cell-to-cell heterogeneity. Our previous work demonstrated that despite heterogeneity, *EGFR*-amplified tumors migrate faster than *EGFR* non-amplified tumors at the population level^11^. Therefore, we sought to determine whether *EGFR* amplification correlated with heterogeneity in individual tumor cell migration patterns. Again using instantaneous speed surface topography plots, we identified significantly more high amplitude peaks for cells tracked in *EGFR*-amplified tumors (Fig. 3A, GBM-8 shown), representing an increased percentage of fast migrating cells when compared to non-amplified cells (Fig. 3B, GBM-5 shown). Across all tumors, these data support an association between *EGFR* amplification and fast migratory behavior of cells, which contributes to overall tumor invasiveness.

Gefitinib, a known inhibitor of EGFR signaling, selectively decreases the overall effective migration speed of *EGFR*-amplified tumors^11^. This prompted us to investigate whether targeting EGFR with gefitinib alters migration parameters within slice cultures on a per cell basis (n = 6 individual tumor slice cultures). Utilizing surface topography analysis, we observed that gefitinib treatment qualitatively decreased high amplitude peaks only in *EGFR*-amplified tumors (Fig. 3C,D). Quantitatively, there was no change in the overall number of observed cell movements. However, we observed a nearly 50% decrease in the frequency of high-amplitude peaks per cell (over 20 μm/hr) in gefitinib treated *EGFR*-amplified tumors, suggesting this molecule can effectively slow migration of fast moving cells (Fig. 3E,F). In amplified tumors, we identified a significant decrease in the standard deviation of instantaneous speed and in the distance traveled by each tumor cell, per peak, with no significant effects observed in non-amplified tumors (Fig. 3G,H). Combined, these data indicate that gefitinib blocks an EGFR dependent bursting behavior present within a subset of migratory tumor cells.

Within *EGFR-*amplified tumors, cells possess varying levels of receptor amplification and EGFR receptor activation^27,28^, which may explain in part the observed heterogeneity in migratory behaviors. To assess heterogeneity across samples, we plotted individual cells, from either EGFR-amplified or non-amplified tumors, as points defined by two behavioral characteristics of migration. Displacement was used to capture the effective migration potential of the cell, and standard deviation of instantaneous speed served as a surrogate of the cell’s bursting behavior. Inhibition of EGFR reduced fast bursting behavior in a subset of cells, thus, we hypothesized that overall population heterogeneity based on these migration parameters would decrease accordingly with gefitinib treatment.

**Figure 4:**
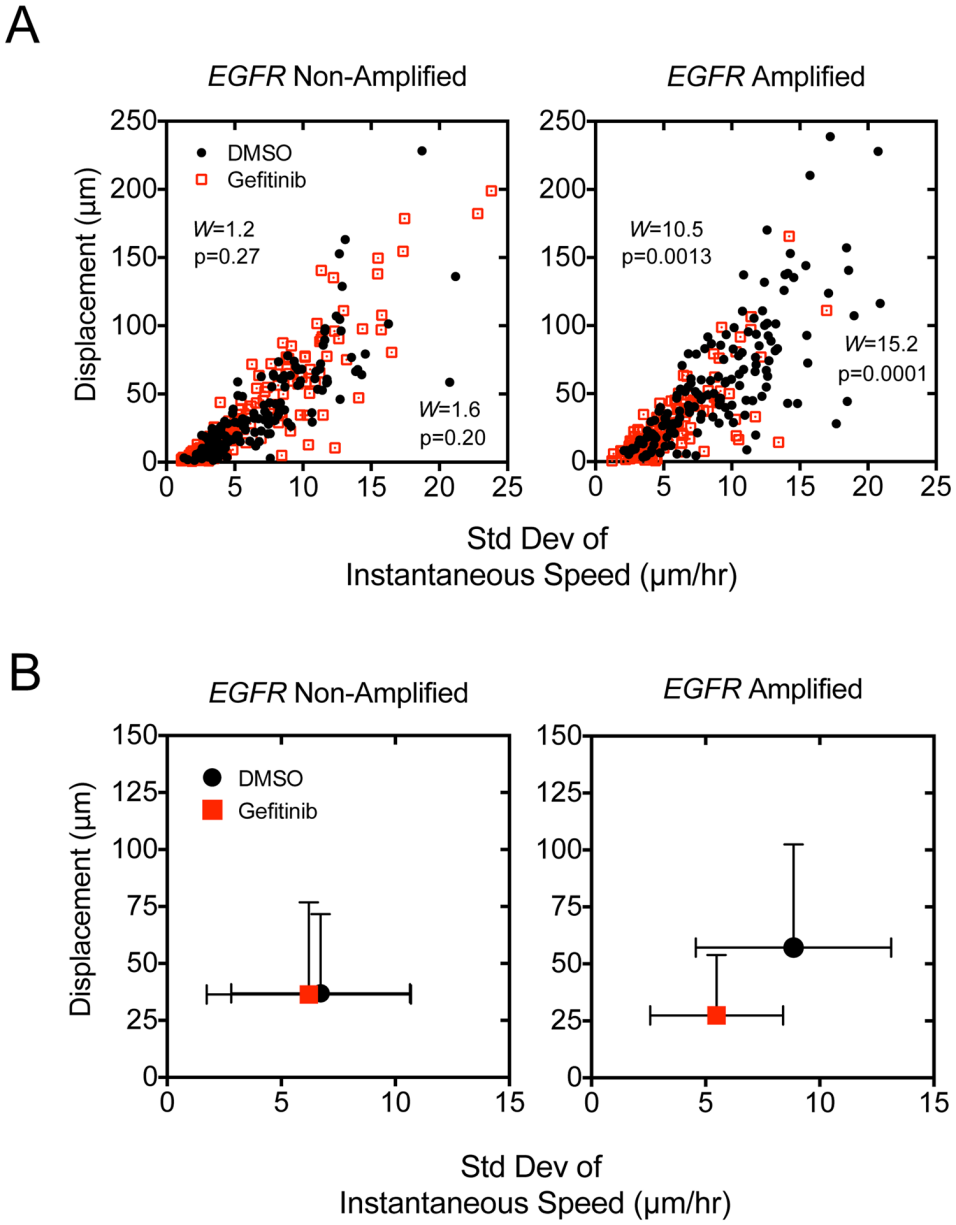
Gefitinib treatment selectively disrupts *EGFR*-Amplification migration behaviors at the tumor cell population level. High temporal resolution cell path tracking occurred every 11 minutes in *EGFR*-amplified (n = 3) and non-amplified tumors (n = 3). Cells from were pooled across tumors for analysis under control (152 *EGFR*-amp cells, 162 non-amp) and gefitinib (10 μM) treatment conditions (129 *EGFR*-amp, 163 non-amp), and were grouped based on tissue level receptor amplification status of the originating tumor. Error bars represent standard deviation (SD). (**A**) Individual cells are displayed according to displacement in micrometers (i.e. total distance traveled during imaging) and standard deviation of instantaneous speed (a metric of an individual cell’s speed variability at anytime during migration) from receptor non-amplified slices (left) demonstrate minimal change in migratory properties despite gefitinib treatment. A dropout of cells with efficient and increased bursting behavior is observed in *EGFR*-amplified slices (right). (**B**) Cell population centroids (bars represent standard deviation) are coincident in receptor non-amplified tumors (left) whereas the population centroid moves to the lower left in amplified tumors (right) after gefitinib treatment.

In both *EGFR*-amplified and non-amplified tumors there was a linear relationship between displacement and standard deviation of instantaneous speed, again suggesting that cell bursting behavior contributes to effective migration. However, the distribution of cells in *EGFR*-amplified tumors was elongated, with more cells falling in the right upper quadrant (both high effective speed and high amplitude movement bursts), representing an augmented subset of cells with enhanced migration potential. In non-amplified tumors treated with gefitinib there was no obvious change in the shape of this distribution, or the centroid position of the population (defined by the mean values for each parameter) (Fig. 4A,B). In striking contrast, treatment of *EGFR*-amplified tumors with gefitinib induced a visible downward shift in the shape of the distribution and in the centroid position (Fig. 4A,B). The change in heterogeneity was assessed using Levene’s test for variance equality. In *EGFR*-amplified tumors, the variance of displacement was significantly decreased (Levene’s statistic, 10.5, p = 0.0013) along with the standard deviation of instantaneous speed for each cell (15.2, p = 0.0001). However, among non-amplified tumors there was no significant change in the variance of displacement (Levene’s statistic 1.2, p = 0.27) or standard deviation of instantaneous speed (1.6, p = 0.20).

As predicted, cells from *EGFR-*amplified tumors appear more heterogeneous in their baseline migratory behavior, with an expansion in the sub-population of migrating tumor cells that travel the farthest and fastest. The presence of this key subpopulation is selectively decreased by gefitinib treatment, supporting the notion that drugs targeting a single pro-migratory pathway in GBM, may have limited effects on the overall population of tumor cells. Although, experiments to confirm that the subpopulation of cells harboring amplification of the *EGFR* locus corresponds specifically to the affected subpopulation of highly invasive cells are underway, our data highlight the potential relevance of personalized therapeutics based upon patient-specific genetic alterations.

## Discussion

An increasing wealth of *in vitro* and *in vivo* data from human tissue describes glioblastoma as a paradigm of continually evolving heterogeneity at the cellular, genomic, transcriptomic, and proteomic levels^14,28–30^. Thus, highlighting a widely supported mechanism underlying treatment resistance and disease recurrence that plagues GBM patients. Efforts to trace the evolution of GBM reveal that many mutations present in the initial tumor are not present in secondary tumors and satellite lesions^31,32^. Not surprisingly, population level molecular characterization of primary GBM tumors, which is now widespread, has little impact on clinical treatment plan or improving patient prognosis.

Even as our understanding of molecular heterogeneity grows, tumor cell invasion remains a barrier to effective treatment in all patients, and no approved therapeutics limit this behavior. Our GBM slice culture model focuses on observation of these clinically important invasive behaviors within the native tumor microenvironment. For the first time, we present dynamic data demonstrating intrinsic heterogeneity in tumor cell migration within living human GBM tissue, which is characterized by juxtaposition of migratory and non-migratory cells, along with variations in speed, directionality, and temporal bursts of increased migration.

We suspect heterogeneity in migratory behavior is a manifestation of the evolving genomic landscape as GBM progresses, and thus could influence clinical prognosis and therapeutic efficacy. Cells within a given GBM population often harbor amplification of up to 3 oncogenic receptor tyrosine kinases, but the majority of cells demonstrate mutually exclusive receptor amplification^17,18,33^. While amplification and mutation of the *EGFR* locus is the most common genetic alteration in GBM^8,34^, recent phylogenetic analysis of primary tissue suggests this may be a late-occurring change^32^, enriched within cells near the infiltrative tumor edge or within satellite lesions^9,10,33^. These data suggest EGFR may drive invasion but not gliomagenesis. Indeed, exogenous overexpression of WT-*EGFR* in oligodendrocyte precursor cells *in vivo* is sufficient to drive cell motility, but insufficient for reliable tumorigenesis^35^.

Human tumors harboring *EGFR*-amplification are more invasive and demonstrate increased overall migration speed in slice cultures^11^. Our current study reveals a small subpopulation of cells within *EGFR*-amplified tumors, characterized by high mean speeds driven by bursting behavior. These short periods of movement correlate with more effective migration, as measured by directionality and displacement, and contribute to widespread heterogeneity in migratory parameters. Within *EGFR*-amplified tumors, not all cells harbor receptor amplification, therefore we postulated increased EGFR gene dose supports augmented cell migration capability. We were unable to directly confirm *EGFR* copy number in a per cell fashion with live imaging. However, we observed that upon slice culture treatment with gefitinib, displacement and peaking behavior are homogenized. After treatment, the centroid of the *EGFR*-amplified population moved, roughly approximating the location of the *EGFR* non-amplified population centroid, suggesting a return to a “baseline” migratory behavior. We believe this represents a selective effect on *EGFR*-amplified cells, as *EGFR* non-amplified cells likely exhibit minimal response to gefitinib treatment.

To effectively design therapeutics limiting GBM invasion, we must understand the molecular mediators underlying heterogeneity in migratory behavior. This study suggests that targeting EGFR in human GBM slices selectively inhibits migration of receptor-amplified GBM cells, resulting in homogenization of the migratory behavior of the population. This is supported by the observations of early effects on the tumor cell speed of tumors within minutes to 1 hour (Supplemental Fig. 2). Although we did not quantitatively assess changes in proliferation rate or cell death, other studies targeting EGFR within *in vivo* glioblastoma models resulted in minimal effects on proliferation, and a predominant effect on tumor invasion^36^. In this *in vivo* study, tumors quickly resumed their infiltrative behavior upon treatment withdrawal, indicating that EGFR inhibition was not sufficient to kill invasive cells, but instead transiently slowed invasion^36^.

While the EGFR pathway appears a promising target for reduction of migration, many parameters remain unexplored. Among studies showing similar bursting migratory behavior in GBM cells^19–23^, some suggest this movement pattern is dependent on the molecular motor myosin II^21^. This implicates myosin II and its regulators as physiologically relevant targets to limit invasion of GBM cells with augmented migratory capabilities^37^. Enhanced signaling through other RTKs may also contribute to heterogeneity^23,34,38^, and these pathways may change over the course of tumor progression. Independent of the chosen target, factors such as optimal dose, penetration of the brain *in vivo*, and the potential need for long-term treatment must be considered.

Although controversial, aggressive strategies including supratotal^39,40^ and fluorescence–guided tumor resection^41^ as well as wide-field FLAIR-based radiation boosting^42^ have recently demonstrated a small but limited potential to control the most infiltrative component of GBM. However, a long-term, patient-specific therapeutic agent that effectively controls GBM infiltration is desperately needed. By focusing on the reduction of observable, clinically important behaviors in actual GBM tissue, our model provides an ideal platform for pinpointing the molecular pathways that lead to disease spread and recurrence. Our hope is that similar studies using the slice culture model will refine our approach to developing and choosing treatments, allowing us to predict and overcome possible mechanisms of resistance within subsets of tumor cells. Though *in vivo* experiments are necessary, this proof of principle study, demonstrates inhibition of a key node in the molecular migration pathway, homogenizes migratory behavior, representing an important step toward improved treatment for GBM patients. Further, *ex vivo* imaging of organotypic slice cultures to quantitate cell behavior responses to candidate therapies is a promising strategy to develop personalized therapy across the spectrum of human cancers.

## Materials and Methods

### Organotypic tumor slice culture preparation

Human glioblastoma tumor tissue was acquired with informed consent by all patients or their legally authorized representative, under an IRB approved protocol at the University of Colorado Hospital. All subsequent experiments were performed in accordance with IRB regulations. Human glioblastoma tissue from contrast enhancing regions was obtained intraoperatively utilizing surgical navigation technology^43–45^. Slice culture generation is described elsewhere^11,46^. Briefly, tumor tissue was embedded in low melting temperature agarose (Invitrogen) and sliced 300–350 μm thick using a VT100S Vibratome (Leica). Tumor slices were cultured on PTFE inserts (Millipore) maintained in a humidified incubator (37 °C and 5% CO_2_) using slice culture medium as described elsewhere^11^. Pathology reports for patient samples (n = 7) provided population-level EGFR-amplification binary scoring, but did not assess levels in individual cells. EGFR amplification status was assessed via fluorescence *in situ* hybridization performed and scored by an accredited cytogenetics laboratory at the University of Colorado Hospital. All samples were IDH1 wild-type (assessed by IHC) and EGFRviii status was not assessed. Individual patient demographic and tumor genetic information is summarized in Supplemental Table 1.

### Retroviral infection of slice cultures

Actively proliferating cells were labeled with ZsGreen1 (Clonetech) via transduction of slice cultures with MMLV-based retroviral particles (10^4^ viral particles/uL) in unsupplemented Neurobasal medium added dropwise to the surface of the tissue slice, 11 days after generation. Viral production is described elsewhere^11,46^. Slices were imaged 72 hours after infection.

### Time-lapse laser scanning confocal imaging of organotypic human tumor slices

Imaging was performed as previously described^11,46^, using a stage-top incubator (Pecon) maintained at 37 °C and 5% CO_2_. Tissue was imaged with a 488 nm laser and 10x air objective (c-Apochromat NA0.45) on a Zeiss LSM510. Imaging fields spanned a 900 μm by 900 μm region between the center and slice edge, and depth varied from 150–250 μm, with a 10 μm Z-step and 11 minute interval between field-scans.

### Tumor cell migration tracking and processing

Two-dimensional maximum intensity projections were generated from three-dimensional Z-stacks using Zeiss Zen software (Zeiss Inc.) Manual cell-tracking was performed by one observer (J.J.P.) marking the centroid of the tumor cell body. Cell position was recorded every 11 minutes using ImageJ (NIH) and MTrackJ^47^. Data analysis was performed on cell tracks with high- (11 minute intervals) or low-temporal resolution (55 minute intervals) using the Chemotaxis and Migration Tool (Ibidi) to calculate mean migration speed (μm/hr), instantaneous migration speed (μm/hr), total path length (μm), and net path length (μm). Directionality, was calculated as the ratio of net path length (displacement) to total path length (μm). Mean effective speed, was calculated as the product of mean cell speed and directionality. Inherent in the transformation of 3-D to 2-D tracking data, all calculated distances and subsequent speeds underestimate actual values.

### Cell track analysis and visualization

Individual cell tracks were analyzed utilizing Prism 6 (GraphPad) to calculate the standard deviation of the instantaneous speed (ratio of the distance traveled to length of the imaging interval). Movement peaks were defined as an instantaneous speed(s) above 0 μm/hr, with at least two adjoining data points, and number of peaks was calculated for each cell using the area under curve function. A secondary metric, “high-speed” peaks, was defined as instantaneous speed greater than 20 μm/hr. We visualized the cell track, time, and instantaneous speed in the x, y, and z axis, respectively, by rendering three-dimensional surface maps (MATLAB) to evaluate cell movement topography. The functional contribution of each peak to overall migration of the cell, was determined by calculating distance traveled per movement, as defined by the area under the curve for each peak.

### Human tumor slice culture gefitinib treatment

Slices were imaged for 11 hours under DMSO (1:1000) control conditions, followed by 11 hours of 10 μm gefitinib (Iressa, Tocris). Between imaging periods slice culture inserts were briefly removed from the stage incubator, media was exchanged with temperature and CO_2_ equilibrated gefitinib containing media, and the exact imaging field re-located. Identical imaging parameters were set for both imaging periods.

### Statistical analysis

Statistical analyses were performed using Prism 6 (GraphPad) and data is represented as mean ± SEM unless otherwise noted. Two-tailed t-tests were used for comparison of two groups of means, while Spearman’s correlation was used to assess the correlation between two individual cell parameters (non-Gaussian distributions; r- and p-values reported). MATLAB R2010a (MathWorks) was used to perform Levene’s test for the equality of variances where appropriate.

## Electronic supplementary material

Supplementary Information

## Data Availability

Raw video files and coordinate based cellular path tracking data for all experiments are available upon request.
