# Supplementary material for: Intratumoral heterogeneity of endogenous tumor cell invasive behavior in human glioblastoma
Source: Sci Rep. 2018 Dec 20;8:18002. doi: 10.1038/s41598-018-36280-9 (PMC6301947; doi:10.1038/s41598-018-36280-9)
Supplement: Supplementary file 1 — Supplementary Information [file 41598_2018_36280_MOESM1_ESM.pdf]

Supplementary Information

Intratumoral heterogeneity of endogenous tumor cell invasive behavior in human glioblastoma

Jonathon J. Parker[1], Peter Canoll[2], Lee Niswander[3], BK Kleinschmidt-DeMasters[4,5], Kara Foshay[6,*], and Allen Waziri[6]

[1]Stanford University School of Medicine, Department of Neurosurgery, Stanford, CA
[2]Columbia University College of Physicians and Surgeons, Department of Pathology and Cell Biology, New York, NY
[3]University of Colorado, Department of Molecular, Cellular & Developmental Biology, Boulder, CO
[4]University of Colorado School of Medicine, Department of Pathology, Anschutz Medical Campus, Aurora, CO
[5]University of Colorado School of Medicine, Department of Neurosurgery, Anschutz Medical Campus, Aurora, CO
[6]Inova Neuroscience and Spine Institute, Inova Health Systems, Falls Church, VA.
*corresponding author: kara.foshay@inova.org

Supplementary Information Includes:
Supplemental Figure 1
Supplemental Figure 2
Supplemental Table 1

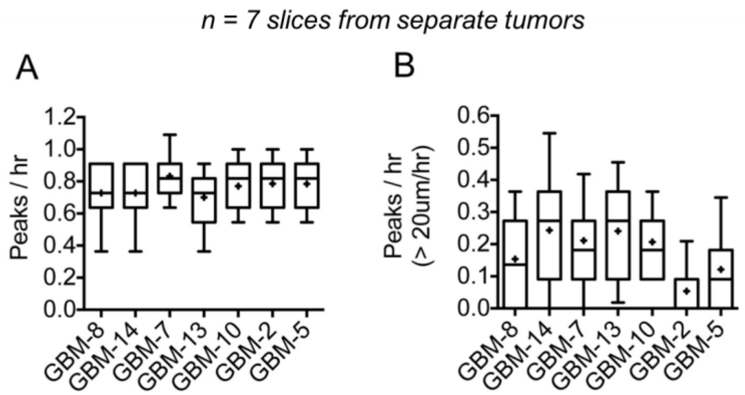

C

	**Number of Cells Tracked at High Temporal Resolution**	
	**DMSO**	**Gefitinib Tx**
GBM-8	58	55
GBM-7	43	29
GBM-13	51	45
GBM-10	55	58
GBM-2	56	51
GBM-5	51	54
	No DMSO	
GBM-14	40	N/A

Supplemental Figure 1. Individual slice cell movement analysis uncovers significant interpatient variation in high-speed peak (“burst”) frequency. A subset of tumor cells (40 to 58, mean 51) were tracked in a group of GBM slice cultures (n=7) with high-temporal resolution (11 minutes). A. The mean total peaks per cell each hour were normally distributed and minimally variable across the tumor cell populations (ANOVA, $p = 0.009$). B. The mean high-speed (>20 µm/hour) peaks per cell each hour varied significantly across the tumor cell populations studied (Kruskal-Wallis ANOVA, $p < 0.001$) ranging from 0.05 to 0.24 peaks per hour. C. The number of cells tracked at high temporal resolution for each slice is represented in tabular form for the current study.

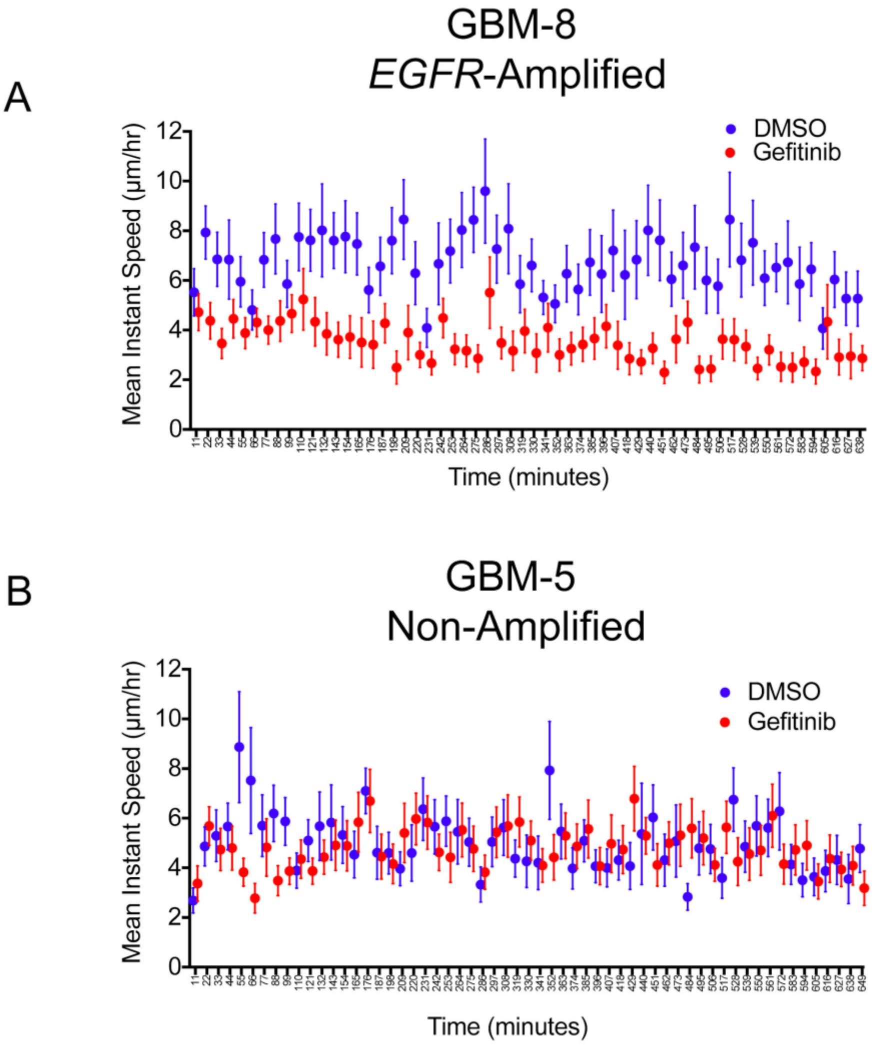

Supplemental Figure 2. EGFR inhibition has an immediate effect on cell migration speed in receptor amplified tumors. Imaging and cell tracking (every 11 minutes) began 15 minutes after addition of media containing 10 µM gefitinib. Approximately 50 cells were tracked in each condition. A. Significant separation existed between the instantaneous speed of the cell population in control (DMSO) versus gefitinib treatment periods in an *EGFR*-amplified tumor (GBM-8) (2-way ANOVA, $p < 0.0001$), and was maintained over 11 hours, with no time dependent effects (2-way ANOVA, $p = 0.80$). B. An *EGFR* non-amplified tumor (GBM-5) shows no response to gefitinib treatment (2-way ANOVA, p 0.18) and no time dependent effects (2-way ANOVA, $p = 0.62$). Error bars represent standard error of the mean (SEM).

**Patient ID**	**Age**	**Sex**	**Pathologic Diagnosis**	**Anatomical Location**	**EGFR Amplification (FISH)**	**IDH1 mutated (IHC)**
GBM-13	55	M	Glioblastoma	Left Temporal	**Y**	**N**
GBM-7	42	M	Glioblastoma	Right Frontal-Parietal	**Y**	**N**
GBM-8	54	M	Glioblastoma (Recurrent)	Left Frontal	**Y**	**N**
GBM-14	53	M	Glioblastoma	Left Temporal	**Y**	**N**
GBM-2	47	M	Glioblastoma (small cell variant)	Left Temporal	**N**	**N**
GBM-5	58	M	Glioblastoma	Right Temporal	**N**	**N**
GBM-10	58	M	Glioblastoma	Right Temporal	**N**	**N**

Supplemental Table 1. Patient demographics and tumor genetics represented in the current study cohort.
